# Misleading presentation of tuberculosis in a child with CGD revealing invasive aspergillosis: A molecularly confirmed case

**DOI:** 10.1016/j.idcr.2026.e02499

**Published:** 2026-01-21

**Authors:** Marjan Aghajani, Nima Parvaneh, Shahram Mahmoudi, Mahmoud Khansari, Fuad Haghighat, Kimia Kamali Sarvestani, Romina Ghazi Mirsaid, Hasti Kamali Sarvestani

**Affiliations:** a Division of Allergy and Clinical Immunology, Department of Pediatrics, Tehran University of Medical Sciences, Tehran, Iran; b Department of Parasitology and Mycology, School of Medicine, Iran University of Medical Sciences, Tehran, Iran; c Department of Surgery, Sina Hospital, Tehran University of Medical Sciences, Tehran, Iran; d Department of Medical Mycology, School of Public Health, Tehran University of Medical Sciences, Tehran, Iran; e Department of Molecular and Cellular Biology, Zand Institute of Higher Education, Shiraz, Iran

**Keywords:** Chronic granulomatous disease, Pneumonia, *Aspergillus fumigatus*, *Aspergillosis*

## Abstract

**Background and Purpose:**

Patients with chronic granulomatous disease (CGD) are susceptible to serious infections including, invasive aspergillosis (IA), which remains a major cause of morbidity and mortality. The diagnosis and management are often challenging due to overlapping clinical features and variable treatment responses.

**Case report:**

Here we report a 4-year-old girl with CGD who admitted with cellulitis, a chest wall abscess, and fever. She had a history of recurrent pneumonia since infancy and a prior diagnosis of tuberculosis (TB). Despite broad-spectrum antibacterial, her condition did not improve. Direct microscopic and macroscopic investigations revealed fungal infection with *Aspergillus* species.

**Conclusion:**

Molecular identification confirmed the isolates as *A*. *fumigatus.* According to the antifungal susceptibility testing, amphotericin B and posaconazole demonstrated strongest activity and the patient was successfully treated by liposomal amphotericin B (50 mg/day) and caspofungin (35 mg/day).

## Introduction

Chronic granulomatous disease (CGD) is a rare inborn error of immunity (IEI) resulting from mutations in the encoding genes of phagocyte enzymes, leading to a defect in the respiratory burst pathway. Consequently, phagocytic cells cannot kill certain microorganisms [1], [2]. The disease can be inherited in two genetic forms, X-linked and autosomal recessive. Although the X- linked form is considered the most common **type globally, data from Iran and other countries in the Middle East and North Africa indicate that the autosomal recessive form** is more prevalent in these regions. [3], [4]. Patients usually develop opportunistic bacterial and fungal infections early in life, with granulomatous inflammation affecting multiple organs [5]. Invasive fungal infections (IFI) are one of the most common causes of morbidity and mortality in patients with CGD. *Aspergillus* species, with the dominance of *A. fumigatus* are important agents of IFI. This species is often responsible for pneumonia, which may disseminate to the liver or brain and can be life-threatening if not promptly diagnosed [6], [7], [8]. Here, we report a case of invasive aspergillosis due to A. fumigatus in a CGD patient, highlighting the diagnostic and therapeutic challenges associated with this rare immunodeficiency.

## Case presentation

**Fig. 1:**
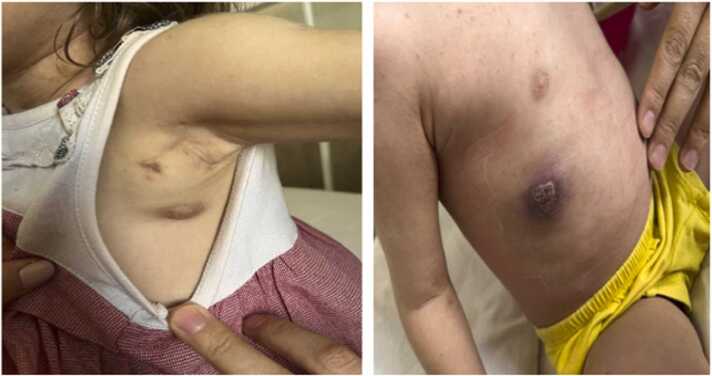
A 4-year-old Iranian girl presenting to the Children’s Medical Center (CMC) in Tehran, Iran, with clinical manifestations of cellulitis, and chest wall abscess.

**Fig. 2:**
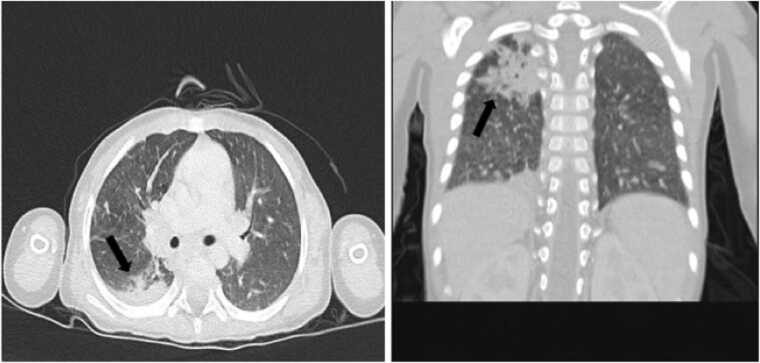
Chest HRCT showing right-lung alveolar consolidation with necrotic lesions and multiple nodules, along with mediastinal and left axillary calcified lymphadenopathy suggestive of TB.

A 4-year-old Iranian girl with the clinical manifestations of cellulitis, a chest wall abscess, and fever presented to the Children Medical Center (CMC) in Tehran, Iran (Fig. 1). She was born to consanguineous parents and her mother had a history of miscarriage. CGD had been confirmed at the age of two, based on genetic testing (nitro blue tetrazolium). The patient had experienced several admissions to the health care centers for the recurrent pneumonia beginning at 21 days of age followed by a diagnosed tuberculosis (TB). As shown in Table 1, during her hospitalization, laboratory findings revealed an anemia (hemoglobin: 10.4 g/dL) with high levels of C-reactive protein (CRP, 21 mg/dl), erythrocyte sedimentation rate (ESR, 60 mm/hour), Lymphocytes (53.8 %), Monocytes (12.5 %) and neutrophil (30.2 %). Moreover, the patient underwent medical imaging of the chest, brain, abdomen and pelvic. Chest radiography and high-resolution computerized tomography (HRCT) demonstrated alveolar consolidation with necrotic lesions in the right lung lobes and multiple nodules of varying sizes. Mediastinal and left axillary adenopathy with calcification were also seen, suggesting TB. No abnormalities were found in other organ systems (Fig. 2). Accordingly, the patient received meropenem (120 mg IV every 8 h), vancomycin (180 mg IV every 6 h), cotrimoxazole (5cc every 12 h), and itraconazole (100 mg/day) but showed no improvement.

**Table 1:** Summary of Laboratory Diagnostic Findings.

Laboratory parameters	Measurement
Hemoglobin	10.4 g/dL
RBC*	4.51 × 10^12^/L
WBC*	9.79 × 10^9^/L
MCV*	70.1 fL
MCH*	23.1 pg
Platelets	423 × 10^3^/L
HCT*	31.6.7 %
Neutrophils	30.2 %
Lymphocytes	53.8 %
Monocytes	12.5 %
Eosinophils	2.9 %
Basophile	0.6 %
ESR*	60 mm/hour
CRP*	21 mg/dl

* RBC, Red blood cell; WBC, White blood cell; MCV, Mean cell volume; MCH, Mean corpuscular hemoglobin; HCT, Hematocrit; ESR, Erythrocyte sedimentation rate; CRP, C-reactive protein

**Fig. 3:**
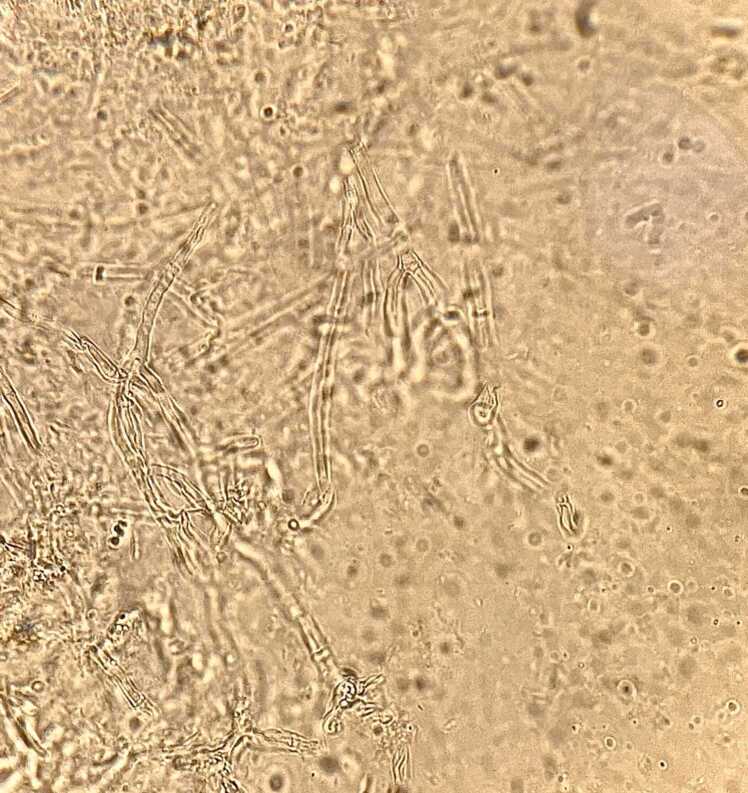
Direct microscopic examination (KOH mount) showing hyaline, septate hyphae with acute-angle dichotomous branching, consistent with Aspergillus spp.

Direct microscopic examination of a wound biopsy revealed branched septate hyphae with dichotomous angels (Fig. 3 A). The specimen was then inoculated on sabouraud dextrose agar (SDA, Merck, Germany) and incubated at 30 °C for up to 12 days. The fungal colonies had a bluish green, broadly spreading with velvet appearance, and the reverse showed a grey pigment which was consistent with *Aspergillus* section *fumigati* (Fig. 3B). For precise identification, genomic DNA was extracted and PCR was carried out for beta-tubulin region using Bt2a (5’-GGTAAC CAA ATC GGT GCT GCT TTC-3’) and Bt2b (5’-ACC CTC AGT GTA GTG ACC CTT GGC-3’) primers. The PCR product was sequenced and the result was analyzed by BLAST (http://www.ncbi.nlm.nih.gov/BLAST) which was compatible with *A. fumigatus*.

Susceptibility of the isolate to itraconazole, voriconazole, amphotericin B and posaconazole (Merck-Germany) was evaluated according to the Clinical and Laboratory Standards Institute CLSI M38-A2 guideline. Based on the epidemiological cut-off values (ECVs), the isolate was wild-type (WT) to amphotericin B (2 µg/mL) and posaconazole (0. 5 µg/mL) and exhibited non wild-type (NWT) phenotype to itraconazole and voriconazole (1 µg/mL). The patient was successfully treated by liposomal amphotericin B (50 mg/day) and caspofungin (35 mg/day) and was discharged. She is now a candidate for hematopoietic stem-cell transplantation (HSCT) and is undergoing necessary pre-transplant evaluations.

## Discussion

CGD is an IEI of the innate immune system, affecting about one in 200,000–300,000 live births worldwide [8]. It was first reported as a “Fatal granulomatous disease of childhood” due to the early mortality of children from opportunistic infections [9]. Accordingly, the median age of diagnosis based on retrospective studies is 6 years in Middle East including Iran [10]. CGD is caused by a defect in the function of the nicotinamide adenine dinucleotide phosphate (NADPH) oxidase complex, which is critical for the production of reactive oxygen species (ROS) during the respiratory burst in phagocytic cells. As a result, due to the lack of ROS production in killing pathogens, patients with CGD are prone to systemic granulomatous inflammations and severe bacterial and fungal infections [1], [2].

IFI is one of the most prevalent infections with high mortality in patients with underlying diseases including CGD. Despite the use of antifungal agents as treatment and prophylaxis, its prevalence ranges from 20 % to 40 % [11]. Non-aspergillus fungal infections are increasingly reported in CGD patients; however, a review of registered cases in CGD patients indicates that IA remains the most common infection, involving the lung, skin, bones, lymph nodes and brain with the dominance of the pulmonary form [3], [12], [13]. In our study, the patient had lung infection with chest wall abscess caused by *Aspergillus* species, which is in line with previous reports [14], [15]. Although based on the available data from Iran, extra pulmonary forms have been frequently observed, which could be due to delayed diagnosis and dissemination of the fungal infections [16], [17], [18], [19]. In this case, the diagnosis of invasive aspergillosis was established through a comprehensive, multimodal diagnostic approach, integrating clinical findings, radiologic features, histopathologic examination, direct microscopy, culture, and antifungal susceptibility testing. Molecular sequencing was applied as a supplementary measure to achieve species-level confirmation and to exclude cryptic or atypical *Aspergillus* species that may not be reliably differentiated by conventional morphologic criteria alone. Importantly, molecular testing was not used as a primary diagnostic tool but rather as an adjunct to conventional mycological methods, which remain central to the diagnosis of invasive fungal infections [20]. This diagnostic strategy reflects routine clinical practice in immunocompromised pediatric populations, where molecular techniques are selectively employed to enhance taxonomic precision and inform antifungal management, particularly in complex cases, without supplanting established diagnostic modalities [21].

The diagnosis of IA is still a challenging problem in patients with CGD due to the overlapping clinical manifestations. These patients may present with few or mild symptoms, often without fever or leukocytosis, even in the setting of severe infection. Moreover, the elevated levels of valuable markers such as ESR and CRP are only useful for the diagnosis of inflammation [22]. Accordingly, the presence of fever and high levels of ESR (60 mm/hour) in our patient were not discriminatory enough to be used for diagnosis of IA.

HRCT scan of the chest is one of the main radiological procedures for diagnosis of IFI and severity of the infection. Based on the European Organization for Research and Treatment of Cancer (EORTC), coexistence of lung consolidations, multiple nodules and vessel occlusion sign (VOS) with or without halo sign are the most common CT features of IA findings [23]. A previous study showed that, in the surveillance chest CT of immunocompromised patients with suspected IFI, 97 of 120 patients (81 %) were confirmed with a 100 % sensitivity and 44 % specificity [24]. However, these findings in children are more variable and can also be seen in TB and other pulmonary infections [25]. In this study, the presence of necrotic nodules with mediastinal and axillary adenopathy suggested misdiagnosis of pulmonary infection caused by TB and no improvement was observed following antimicrobial treatment. Accordingly, confirmation of fungal infections using mycological and molecular methods as the gold standard should be regarded.

Findings from a systematic review indicate that among *Aspergillus* species, *A. fumigatus* followed by *A. nidulans* are the most common species causing IA in patients with CGD [3]. Similarly, in our case, *A. fumigatus* was confirmed by micro- and macro-morphological examination, and PCR-sequencing of the surgical wound biopsy. However, Sugui et al. reported IA caused by *A. tanneri* from two patients with CGD who had primary pulmonary with contiguous involvement of multiple organs, including the liver, spleen, and gastrointestinal tract [26]. In other studies, less common aspergillus species including *A. terreus* and *A. niger* have been isolated from broncho alveolar lavage and bronchial secretions of patients with CGD respectively [27], [28]. Furthermore, in the patient with a previous history of CGD, lymphadenitis and pulmonary involvement caused by *A. flavus* was identified [29].

Management of IA in children with CGD is challenging due to significant differences in pharmacokinetic profile, long-term prophylaxis treatment and resistance to antifungal agents [30]. Previous studies have shown that pharmacokinetic variability of voriconazole as first-line therapy results in variable serum concentrations in pediatric patients. Consequently, they require **higher weight-doses** of agents to achieve therapeutic levels, which increases their susceptibility to **serious drug-related adverse effects** [31], [32]. Furthermore, it is recommended to receive antifungal triazoles such as itraconazole and posaconazole at a younger age to prevent fungal infections in children with CGD [33]. However, prolonged prophylaxis treatment is associated with an increased risk of severe side effects, including hepatotoxicity and resistance to antifungal agents [34]. In recent years, the prevalence of azole-resistance in *A. fumigatus* has increased [35]. Recently, Pfaller et al. reported that 9 %, 3.6 %, and 4.5 % of *A. fumigatus* isolates were NWT to itraconazole, posaconazole and voriconazole according to CLSI method, respectively [36]. Moreover, the high MIC value of amphotericin B has been reported in several studies [37]. However, in the present study, amphotericin B and posaconazole showed good *in vitro* activity against *A. fumigatus* isolate. These results were consistent with previous studies on *A. fumigatus* that suggested the combination of liposomal amphotericin B and echinocandins as a promising treatment in IA [38]. In our study, the isolate exhibited NWT phenotype to itraconazole and voriconazole in consistent with previous report [39]. This finding is clinically concerning, as azole-resistance is a major agent in treatment failure and increased mortality in IA patients with underlying diseases, including CGD. Nevertheless, our patient showed good response to clinical treatment by amphotericin B (50 mg/day) and caspofungin (35 mg/day). Given that HSCT is the principle treatment for CGD patient in early ages [40], she is currently undergoing the necessary pre-transplant evaluations.

## Conclusion

In conclusion, IA remains a major cause of morbidity and mortality among patients with CGD. Considering their inherent immune deficiency, timely and accurate diagnosis of fungal infection, along with treatment guided by antifungal susceptibility testing, is recommended. Fortunately, the present case was treated successfully with amphotericin B and caspofungin and was discharged in stable condition.

## Ethical Considerations

The authors confirm that all the data and figures in this article are original and have not been published elsewhere. All the authors contributed sufficiently to the work and have approved the final version of the manuscript.

## Funding

This research did not receive any specific grant from funding agencies in the public, commercial, or not-for-profit sectors. The funders had no role in the study design; data collection, analysis, or interpretation; manuscript preparation; or the decision to submit the manuscript for publication.

## Author Statement

M.A. and N.P. contributed equally to this work. M.A. and N.P. were involved in the clinical management of the patient and collection of clinical data. S.M. and H.K.S. performed the mycological investigations, including direct microscopy, culture, molecular identification, and antifungal susceptibility testing. M.K. and F.H. contributed to surgical management and histopathological evaluation. K.K.S. assisted with molecular analysis and data interpretation.

## CRediT authorship contribution statement

**Hasti Kamali Sarvestani:** Supervision, Project administration, Methodology, Investigation. **Marjan Aghajani:** Methodology, Investigation. **Shahram Mahmoudi:** Methodology, Investigation. **Nima Parvaneh:** Supervision, Methodology, Investigation. **Fuad Haghighat:** Investigation, Data curation. **Mahmoud Khansari:** Resources, Investigation. **Romina Ghazi Mirsaid:** Writing – original draft, Investigation. **Kimia Kamali Sarvestani:** Software, Investigation.

## Declaration of Competing Interest

The authors declare that they have **no known competing financial interests or personal relationships** that could have appeared to influence the work reported in this manuscript. The authors also confirm that **no conflicts of interest** exist regarding the research, authorship, or publication of this article.
